# Supplementary material for: Communicating risk in human-wildlife interactions: How stories and images move minds
Source: PLoS One. 2020 Dec 28;15(12):e0244440. doi: 10.1371/journal.pone.0244440 (PMC7769453; doi:10.1371/journal.pone.0244440)
Supplement: S1 Research design — (PDF) [file pone.0244440.s001.pdf]

S1 Research design

We worked with i-Link Research Solutions (i-Link), an Australian online survey firm, to field a survey of 3,200 Australians between May 2, 2019 and May 20, 2019. i-Link manages a panel of over 200,000 Australians recruited through a variety of means, including print media, online marketing, direct mail, affiliate partners, and personal invitations. Members of the panel were invited to participate in our survey based on where they lived; we restricted our sample to residents of Queensland and New South Wales who were over 18 years of age.

Prior to assignment to treatment, we identified postcodes with at least one known bat roost. Bat roost locations were obtained from state government websites. Bat roost locations in QLD were obtained from the following website: `https://data.qld.gov.au/dataset/flying-fox-monitoring-program/resource/2079912d-72ac-4116-9e12-08e068064bff`. Bat roost locations in NSW were obtained from the following website: `https://data.nsw.gov.au/data/dataset/grey-headed-flying-fox-camps64935`. To fill in any spatial/temporal gaps in NSW, roost locations known to be inhabited within the last five years were also provided by Peggy Eby, focusing primarily on NSW and areas south of Bowen. S1 Fig is a map depicting postcodes with flying fox roosts and postcodes without flying fox roosts. We block randomized assignment to treatment based on whether or not a participant resided in a postcode with known bat roosts (S2 Table).

Prior Attitudes

We measured attitudes towards bats prior to treatment by asking survey participants the following question: "Please rate how you feel about flying foxes/bats on a feeling

thermometer using a scale of 0 to 100. The higher the number, the warmer or more favorable you feel toward bats. The lower the number, the colder or less favorable you feel. You can pick any number between 0 and 100." The respondent was then instructed to click on a scale to indicate their selection or drag the slider to select their answer.

Treatment

We randomly assigned respondents to one of six conditions: villain narrative without an image, victim narrative without an image, non-narrative information without an image, villain narrative with an image, victim narrative with an image, non-narrative information with an image (Table S1 Table). Conditions are depicted in the style of a Facebook post and presented in S2 Fig.

Affect

To measure affect, we asked survey participants to rate the intensity of their response to the Facebook post, using the following wording: "Upon reading this Facebook post, imagine what emojis you would use to respond. Please rate how intensely you are experiencing each of the following emojis."

Each respondent was prompted to select a number on a scale of 1 (Minimal Intensity) to 10 (Maximum Intensity), or 0 (Not at all), for each emoji. Emojis were labeled with emotions from the Positive and Negative Affect Schedule [62].

The positive affective score is a sum of the intensity ratings for positive emotions: Amused, Inspired, Hopeful, Enthusiastic, Determined, Love, Happy, and Thumbs Up. The negative affective score is a sum of the intensity ratings for negative emotions: Disgusted, Annoyed, Frustrated, Hostile, Anxious, Embarrassed, Fearful, Nervous, Sad, Guilty, Upset, and Thumbs Down. A sample of emojis used is depicted in S3 Fig.

To generate a single affective score, we subtracted the overall negative score from the overall positive score such that a negative affective score corresponds to a respondent whose affective response was more negative than positive and a positive affective score corresponds to a respondent whose affective response was more positive than negative.

Risk Perception

We measured two component parts of risk perception: perceived impact associated with bats and perceived likelihood of impact associated with bats. Impact was coded on a scale of -2 (Extremely negative) to 2 (Extremely positive) and likelihood was coded on a scale of 1 (Extremely unlikely) to 5 (Extremely likely). The impact measure included in the model is an additive index of the six impact questions; the likelihood measure is an additive index of the six likelihood questions.

- Impact
  - We would like to know your thoughts about various impacts flying foxes may have.
    - What is the economic impact of flying foxes to you personally?
      ☐ Extremely negative, ☐ Negative, ☐ Neither negative nor positive, ☐ Positive, ☐ Extremely positive
    - What is the economic impact of flying foxes to your community?
      ☐ Extremely negative, ☐ Negative, ☐ Neither negative nor positive, ☐ Positive, ☐ Extremely positive
    - What is the impact of flying foxes on your personal quality of life?
      ☐ Extremely negative, ☐ Negative, ☐ Neither negative nor positive, ☐ Positive, ☐ Extremely positive
    - What is the impact of flying foxes on your community's quality of life?
      ☐ Extremely negative, ☐ Negative, ☐ Neither negative nor positive, ☐ Positive, ☐ Extremely positive
    - What is the impact of flying foxes on your personal health?
      ☐ Extremely negative, ☐ Negative, ☐ Neither negative nor positive, ☐ Positive, ☐ Extremely positive
    - What is the impact of flying foxes on the health of others in your community?
      ☐ Extremely negative, ☐ Negative, ☐ Neither negative nor positive, ☐ Positive, ☐ Extremely positive
- Likelihood

- Now that you have given us a sense of the impacts flying foxes have on you and your community, now we would like to know how likely you think these impacts will occur within the next year.
  * Have an economic impact on you personally?
    ☐ Extremely likely, ☐ Moderately likely, ☐ Equally likely/unlikely, ☐ Moderately unlikely, ☐ Extremely unlikely
  * Have an economic impact on your community?
    ☐ Extremely likely, ☐ Moderately likely, ☐ Equally likely/unlikely, ☐ Moderately unlikely, ☐ Extremely unlikely
  * Impact your personal quality of life?
    ☐ Extremely likely, ☐ Moderately likely, ☐ Equally likely/unlikely, ☐ Moderately unlikely, ☐ Extremely unlikely
  * Impact your community's quality of life?
    ☐ Extremely likely, ☐ Moderately likely, ☐ Equally likely/unlikely, ☐ Moderately unlikely, ☐ Extremely unlikely
  * Impact your personal health?
    ☐ Extremely likely, ☐ Moderately likely, ☐ Equally likely/unlikely, ☐ Moderately unlikely, ☐ Extremely unlikely
  * Impact human health of others in your community?
    ☐ Extremely likely, ☐ Moderately likely, ☐ Equally likely/unlikely, ☐ Moderately unlikely, ☐ Extremely unlikely

Outcome Variables

We measured survey participant's level of support for five bat management policies. Support was coded on a scale of 1 (Strongly oppose) to 4 (Strongly support).

- To what extent do you support the following:
  - Providing federal protection for flying fox species threatened with extinction
    ☐ Strongly support, ☐ Support, ☐ Oppose, ☐ Strongly oppose
  - Providing state protection for flying fox species threatened with extinction
    ☐ Strongly support, ☐ Support, ☐ Oppose, ☐ Strongly oppose

- Dispersing roosts close to residential areas

  ☐ Strongly support, ☐ Support, ☐ Oppose, ☐ Strongly oppose

- Dispersing roosts in urban public spaces

  ☐ Strongly support, ☐ Support, ☐ Oppose, ☐ Strongly oppose

- Investing in strategic tree plantings outside of urban areas to encourage flying foxes to roost and forage outside urban areas ☐ Strongly support, ☐ Support, ☐ Oppose, ☐ Strongly oppose

Covariates

We collected the following information to include as covariates in our model:

- State
  - Based on respondent's confirmed postcode
- Gender
  - What is your gender?
    - Male
    - Female
    - Other
- Age
  - What is your age?
    - 18-24 years
    - 25-34 years
    - 35-44 years
    - 45-54 years
    - 55+ years
- Education
  - What is the highest level of education you have completed?
    - Primary school

    * Year 7 to Year 9
    * Year 10
    * Year 11
    * Year 12
    * Non-trade qualification
    * Trade qualification
    * Associate Diploma
    * Undergraduate Diploma
    * Bachelor Degree (including Honours)
    * Postgraduate Degree

- Income
  - What is the gross annual income, before tax or other deductions, for you and your family or others living with you from all sources? Please include any pensions and allowances, and income from interest or dividends.
    * Less than $10,000 per year
    * $10,001 to $15,000 per year
    * $15,001 to $20,000 per year
    * $20,001 to $25,000 per year
    * $25,001 to $30,000 per year
    * $30,001 to $35,000 per year
    * $35,001 to $40,000 per year
    * $40,001 to $45,000 per year
    * $45,001 to $50,000 per year
    * $50,001 to $60,000 per year
    * $60,001 to $70,000 per year
    * $70,001 to $80,000 per year
    * $80,001 to $90,000 per year
    * $90,001 to $100,000 per year
    * $100,001 to $110,000 per year

        - $110,001 to $120,000 per year
        - $120,001 to $130,000 per year
        - $130,001 to $140,000 per year
        - $140,001 to $150,000 per year
        - $150,001 to $160,000 per year
        - $160,001 to $180,000 per year
        - More than $180,000 per year

- Exposure to roosts
    - How often do you see flying foxes roosting near where you...

      Note: A flying fox roost is a site where flying foxes congregate during the day for rest.

        - ...Live? ☐ Always, ☐ Sometimes, ☐ Rarely, ☐ Never
        - ...Work? ☐ Always, ☐ Sometimes, ☐ Rarely, ☐ Never
        - ...Recreate? ☐ Always, ☐ Sometimes, ☐ Rarely, ☐ Never

- Exposure to bats foraging
    - How often do you see flying foxes foraging near where you...

      Note: Foraging occurs when flying foxes are searching for and eating food.

        - ...Live? ☐ Always, ☐ Sometimes, ☐ Rarely, ☐ Never
        - ...Work? ☐ Always, ☐ Sometimes, ☐ Rarely, ☐ Never
        - ...Recreate? ☐ Always, ☐ Sometimes, ☐ Rarely, ☐ Never

The following equations correspond to our conditional process model. Figure S4 Fig shows a statistical diagram of the model.

$$M_1 = \gamma_{M1} + \sum_{i=1}^{5} \alpha_i D_i + \alpha_6 W + \sum_{j=7}^{11} \alpha_j D_i W + \sum_{k=1}^{7} \rho_k C_k + \epsilon_{M1} \tag{1}$$

$$M_2 = \gamma_{M2} + \sum_{i=12}^{16} \sum_{j=1}^{5} \alpha_i D_j + \alpha_{17} W + \sum_{k=18}^{22} \alpha_k D_i W + \delta_1 M_1 + \alpha_{23} M_1 W + \sum_{l=8}^{14} \sum_{m=1}^{7} \rho_l C_m + \epsilon_{M2} \tag{2}$$

$$M_3 = \gamma_{M3} + \sum_{i=1}^{5}\sum_{j=24}^{28} \alpha_j D_i + \alpha_{29} W + \sum_{k=30}^{34} \alpha_k D_i W + \delta_2 M_1 + \alpha_{35} M_1 W + \sum_{l=15}^{21}\sum_{m=1}^{7} \rho_l C_m + \epsilon_{M3} \tag{3}$$

$$Y = \gamma_Y + \sum_{i=1}^{5} \zeta_i D_i + \zeta_6 W + \sum_{j=7}^{11} \zeta_j D_i W + \sum_{k=1}^{3} \beta_k M_k + \sum_{l=4}^{6} \beta_l M_k W + \sum_{m=22}^{28}\sum_{n=1}^{7} \rho_m C_n + \epsilon_Y \tag{4}$$
