# Supplementary material for: Communicating risk in human-wildlife interactions: How stories and images move minds
Source: PLoS One. 2020 Dec 28;15(12):e0244440. doi: 10.1371/journal.pone.0244440 (PMC7769453; doi:10.1371/journal.pone.0244440)
Supplement: S1 Results — (PDF) [file pone.0244440.s002.pdf]

S1 Results

As illustrated in Table S4 Table, both the victim and the villain narratives elicit more negative affective responses compared to the non-narrative message, with the villain narrative eliciting the most negative response. The addition of an image to the victim narrative produces a more negative response than the victim narrative without an image. Conversely, the addition of an image to the villain narrative produces a less negative response than the villain narrative without an image.

The villain narrative with image treatment corresponds to the most negative impacts perceived by survey participants of all the conditions. The victim narrative with image treatment corresponds to the highest reported likelihood of impacts perceived by survey participants across all conditions.

Tables S7 Table and S8 Table show the means and standard deviations for the intensity ratings corresponding to each emoji among participants with warm priors and cool priors, respectively. These were calculated by first partitioning participants with warm priors and cool priors from the rest of the sample. Participants with warm prior attitudes towards bats reported attitudes above one standard deviation above the mean. The mean prior attitude was 52 on a scale of 0 to 100, thus we characterize the mean as corresponding with neutral feelings towards bats. Participants with cool prior attitudes towards bats reported attitudes below one standard deviation below the mean. The sample was partitioned only to populate Tables S7 Table and S8 Table; references to participants with warm and cool prior attitudes towards bats in the main text are based on model estimates corresponding to one standard deviation above and below the mean, respectively.

According to Table S7 Table, participants with warm priors who receive the victim narrative treatment report feeling Sad, Upset, and Frustrated, but also give a Thumbs Up, report feeling Hopeful, and Enthusiastic. The mean intensity of the top positive emotions goes down for this subsample of participants when the victim narrative is accompanied by an image, while the top negative emotions are more felt more intensely with the addition of an image. While these individuals feel some positive emotions when presented with the victim narrative, their feelings are overwhelmingly negative when presented with the villain narrative, with Thumbs Down, Sad, Annoyed, Upset, Disgusted, Frustrated, and Hostile averaging above 5 on the intensity scale (0 to 10). The intensities of these negative emotions soften slightly with the addition of an image to the villain narrative.

Participants with cool priors (Table S8 Table) exposed to the victim and villain treatments report feeling Sad, Upset, and Frustrated, but not at the same intensity as those with warm prior attitudes towards bats. Those with cool priors also reported feeling above a 5 average on the intensity scale for Thumbs Up, Enthusiastic, Love, and Hopeful when presented with the victim narrative without and image. The addition of an image dampens some of these emotions. When presented with the villain narrative with an image, these respondents give the Thumbs Up with an average 5.42 intensity, the highest average intensity rating for a positive emotion associated with the villain narrative among those with cool priors.

Overall, those with warm prior attitudes towards bats seem to react more strongly – positively and negatively – to narratives casting bats as victims or villains.
