# Supplementary figures and images for: Communicating risk in human-wildlife interactions: How stories and images move minds
Source: PLoS One. 2020 Dec 28;15(12):e0244440. doi: 10.1371/journal.pone.0244440 (PMC7769453; doi:10.1371/journal.pone.0244440)

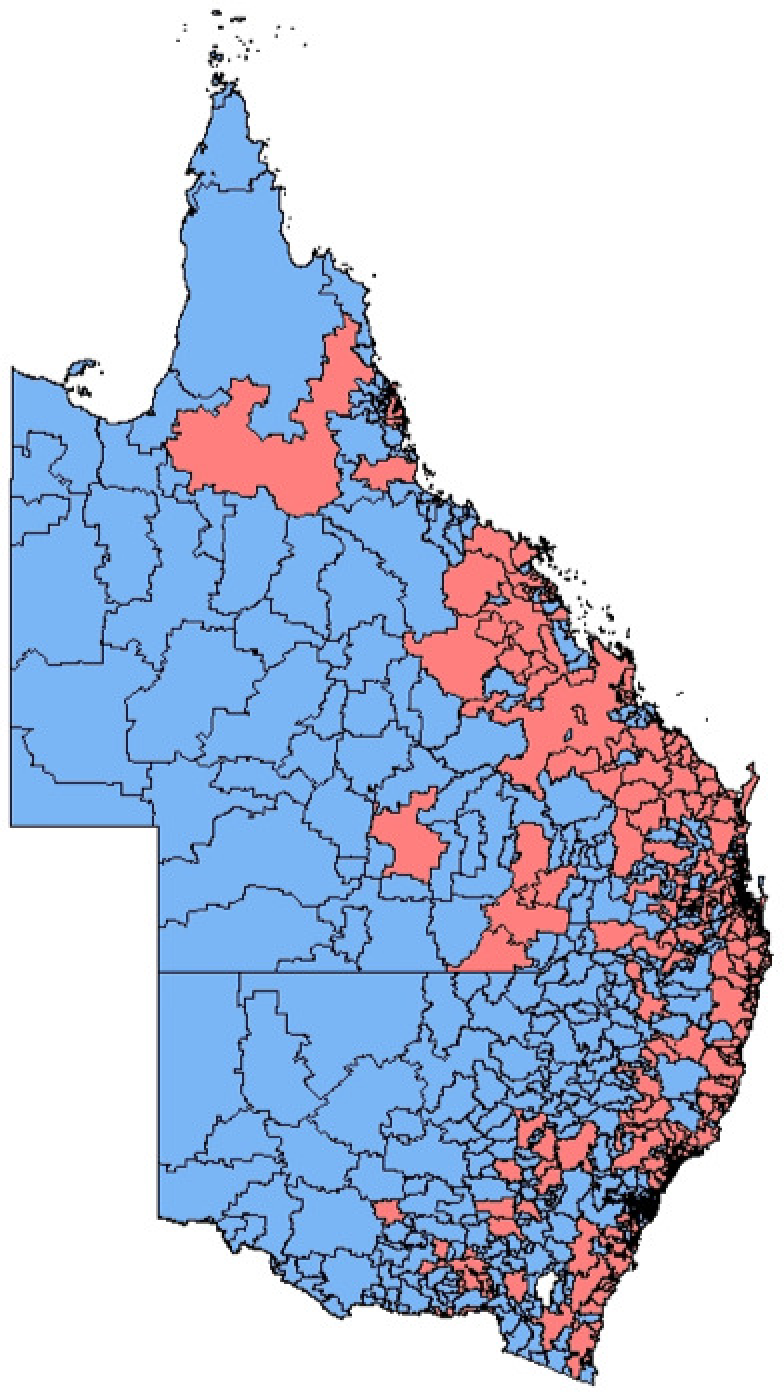

Supplement: S1 Fig — Blue represents postcodes without known bat roosts; pink represents postcodes with known bat roosts. (TIF) [file pone.0244440.s003.tif]

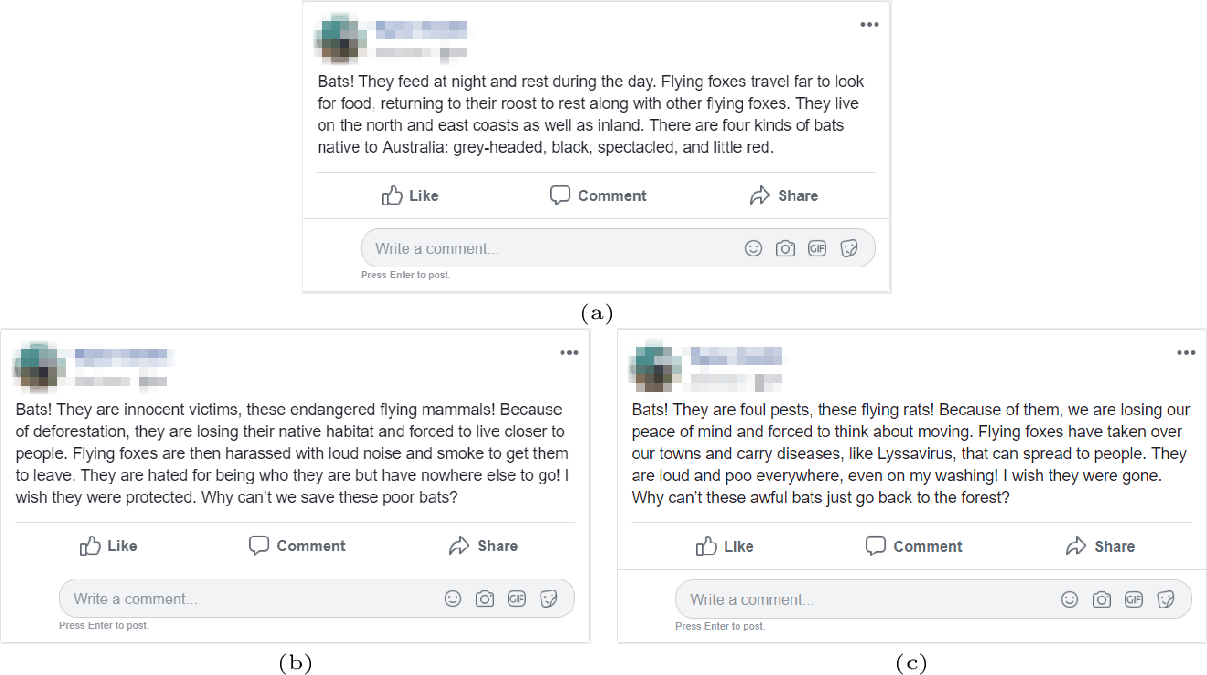

Supplement: S2 Fig — Depiction of non-narrative (a), victim narrative (b), and villain narrative (c) conditions, without image treatment. (TIF) [file pone.0244440.s004.tif]

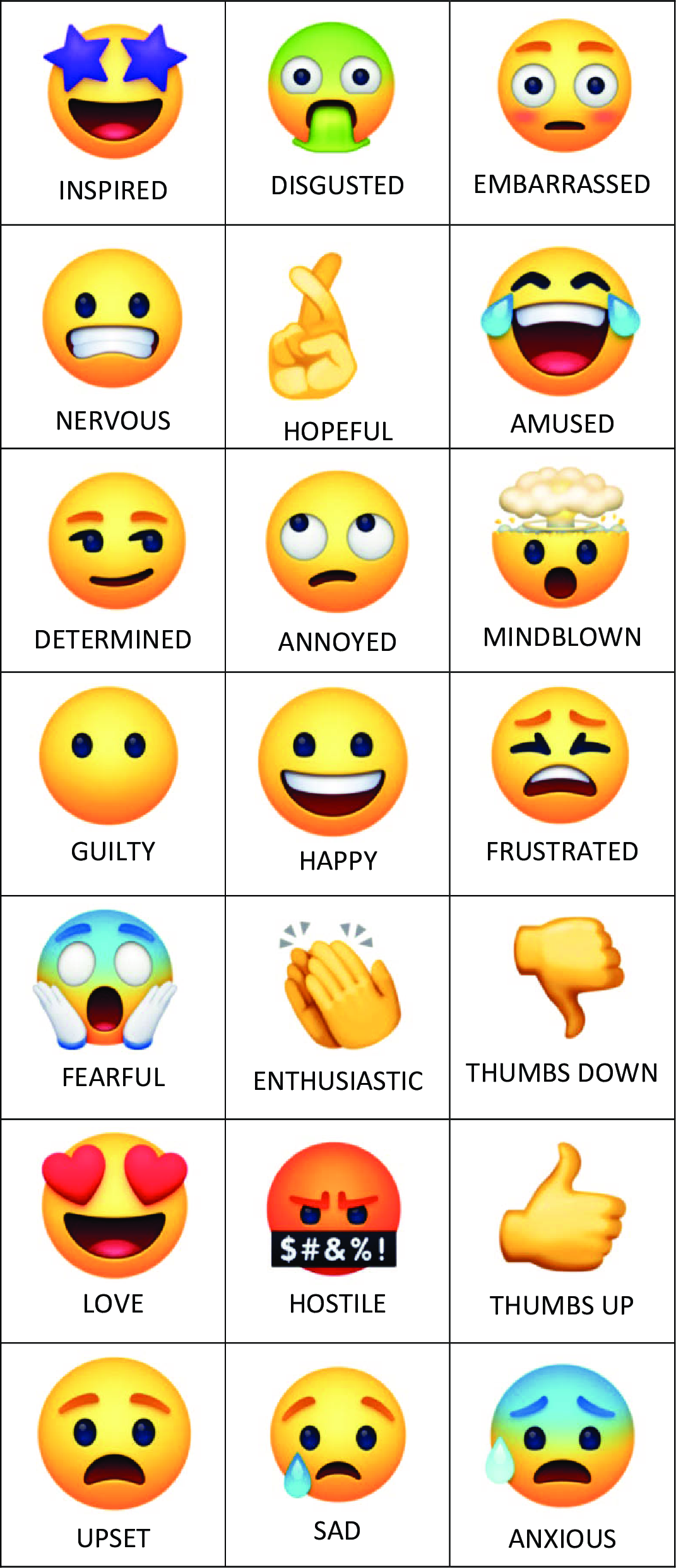

Supplement: S3 Fig — (TIF) [file pone.0244440.s005.tif]

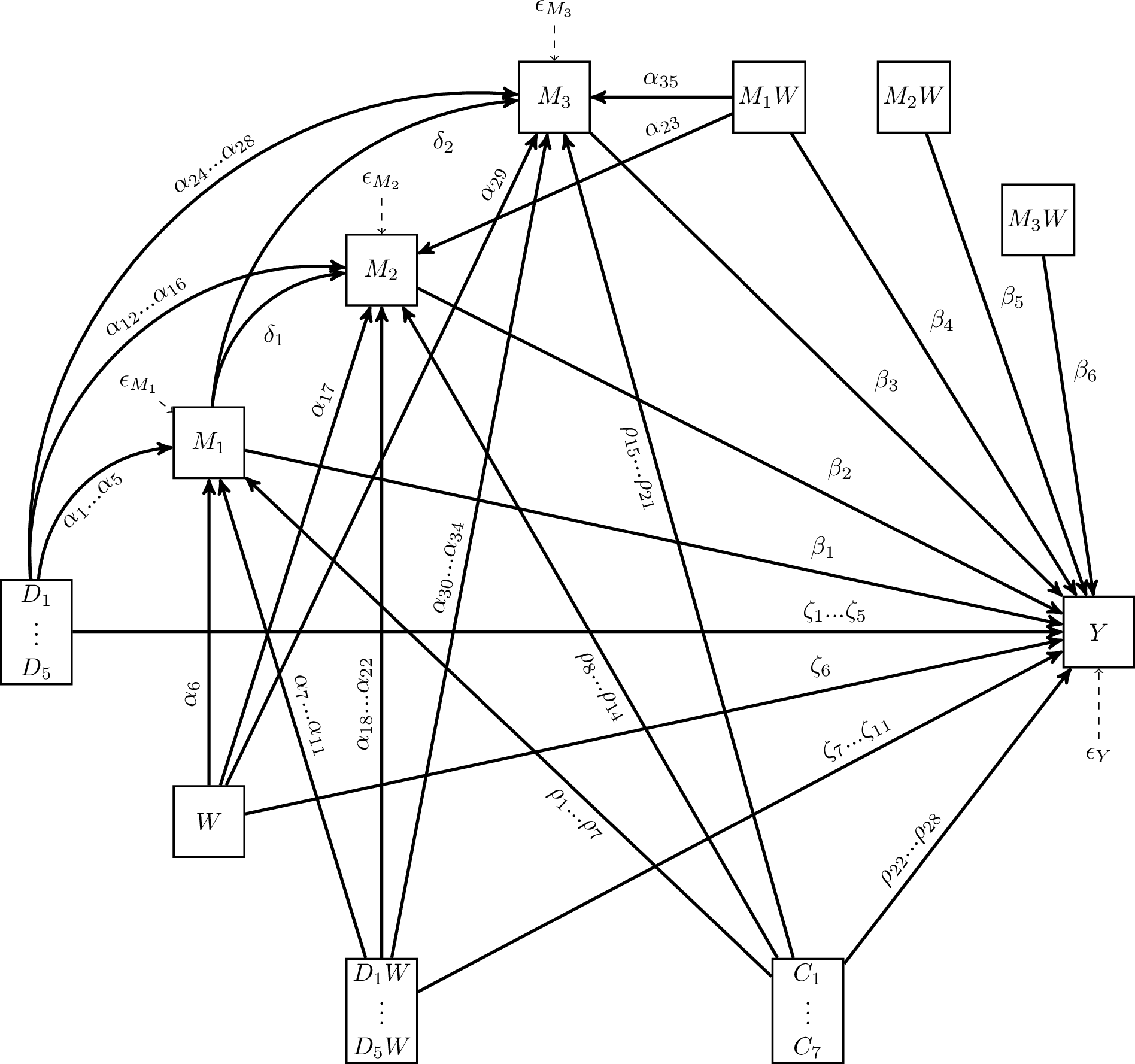

Supplement: S4 Fig — (TIF) [file pone.0244440.s006.tif]

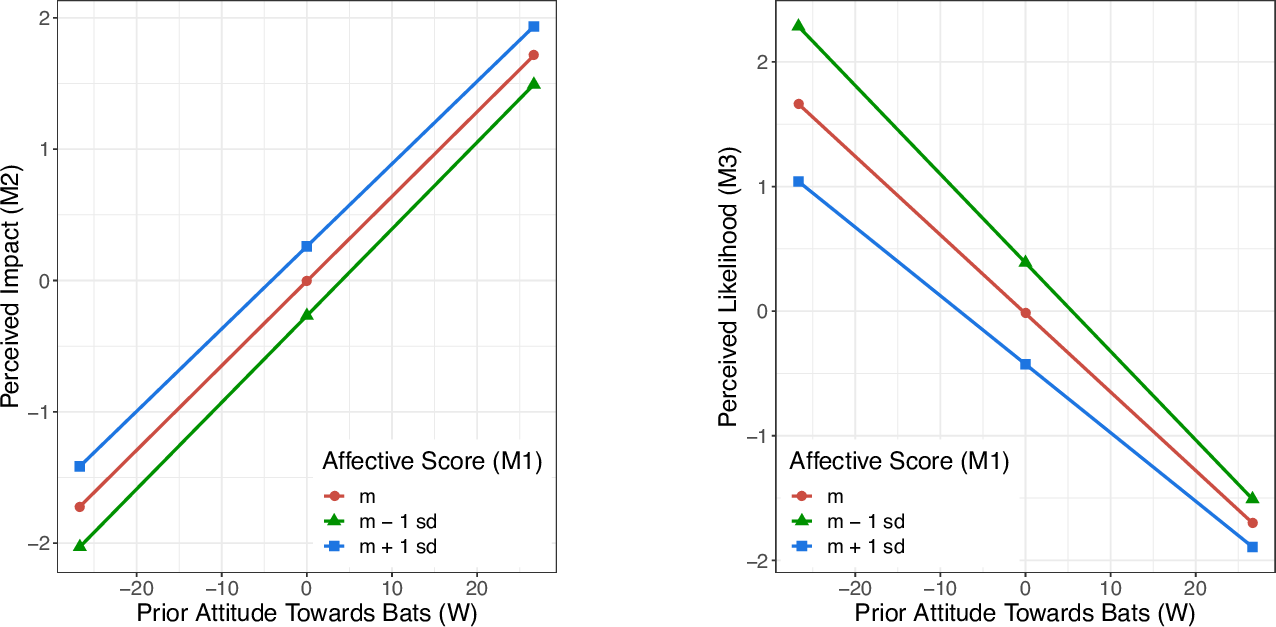

Supplement: S5 Fig — (TIF) [file pone.0244440.s007.tif]

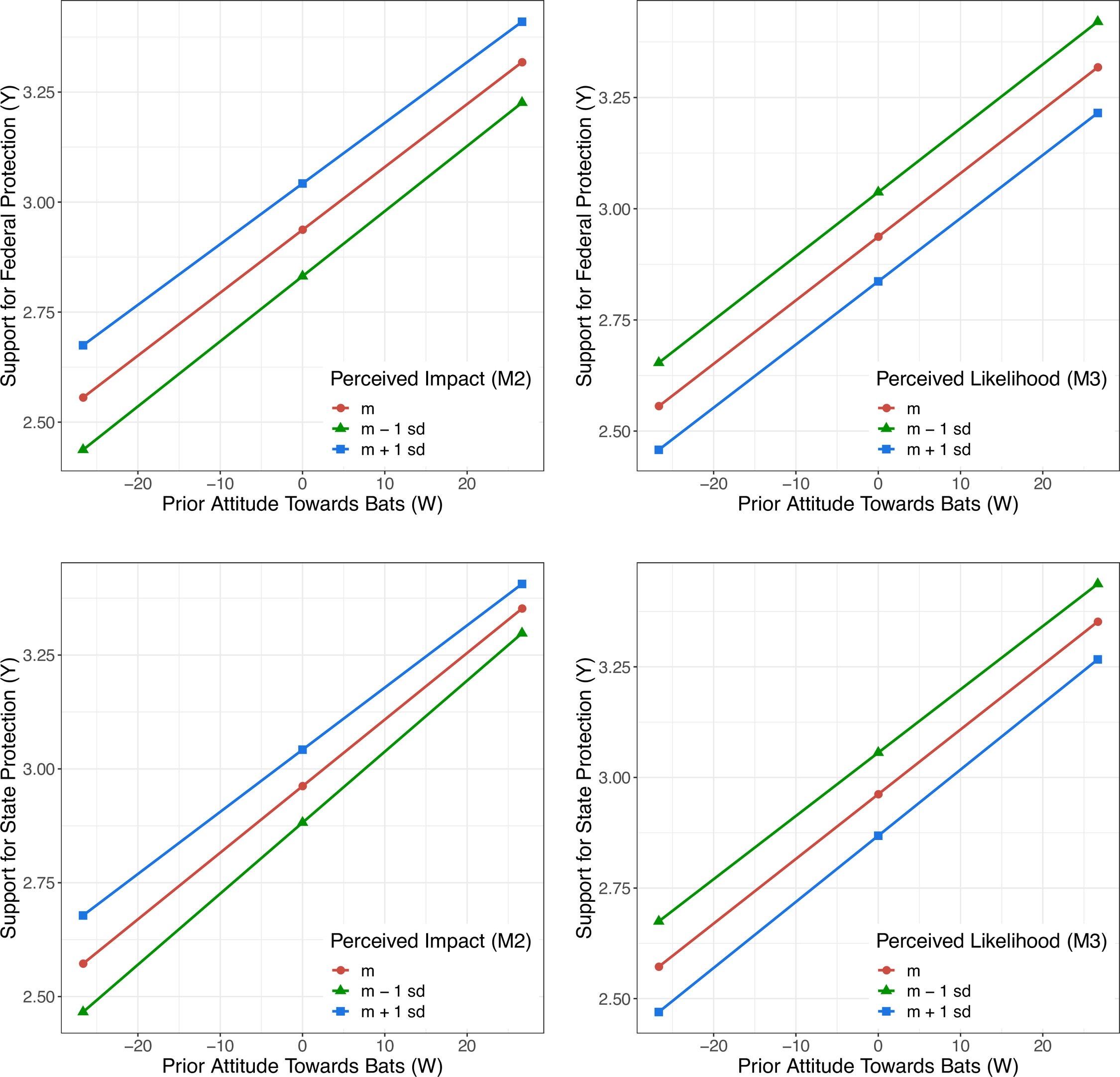

Supplement: S6 Fig — (TIF) [file pone.0244440.s008.tif]

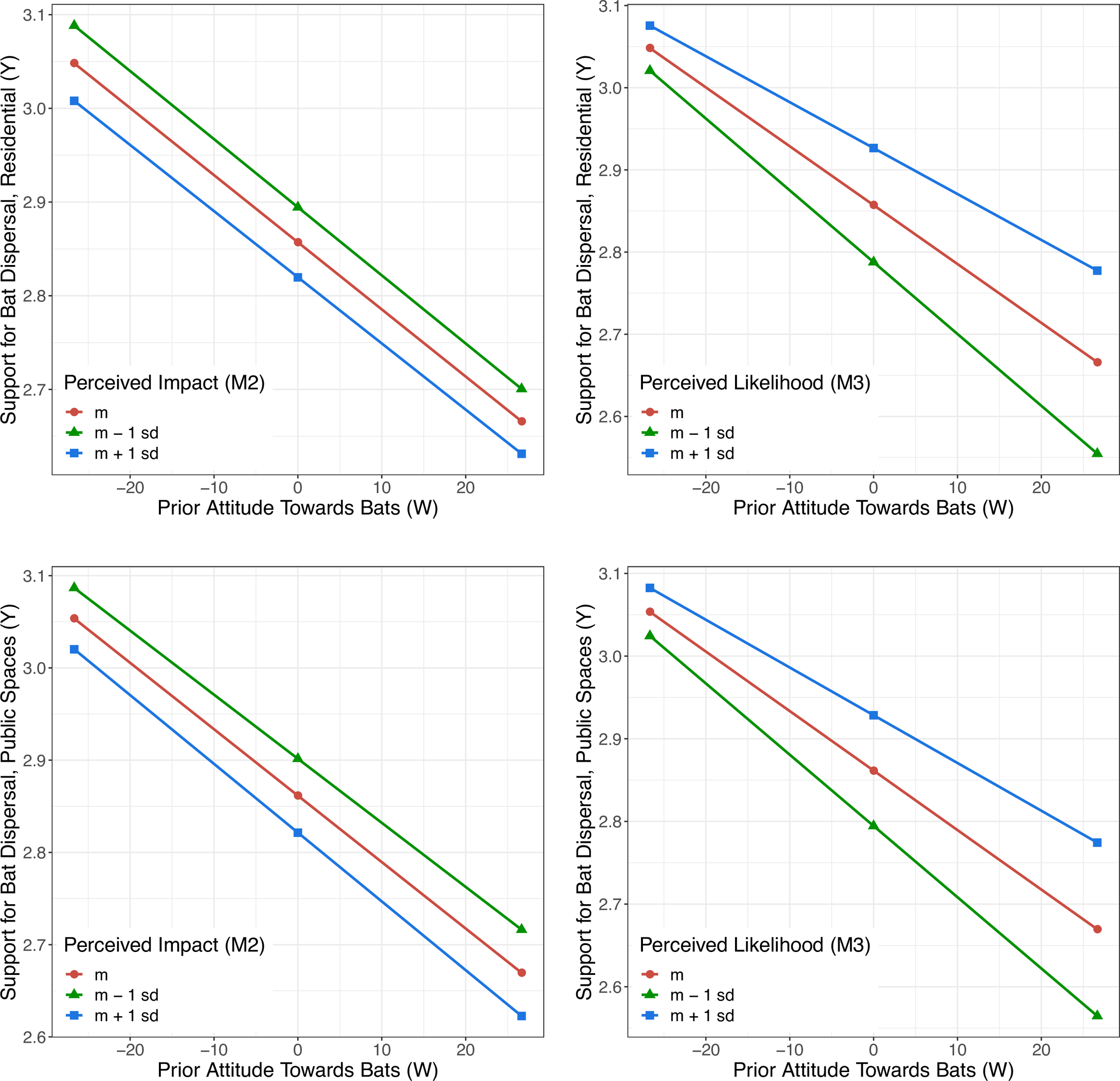

Supplement: S7 Fig — (TIF) [file pone.0244440.s009.tif]

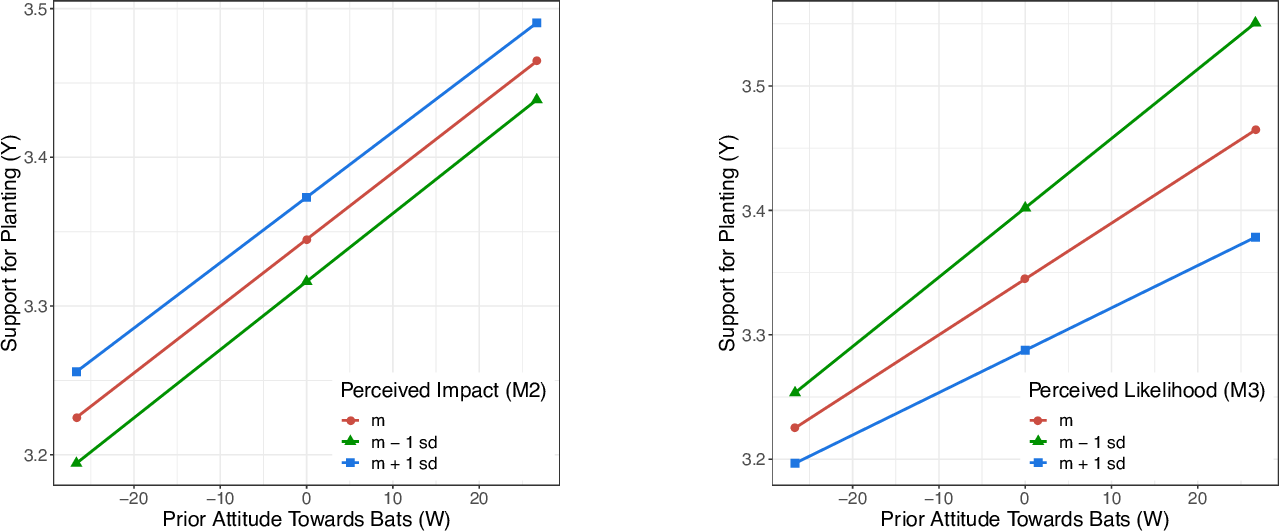

Supplement: S8 Fig — (TIF) [file pone.0244440.s010.tif]

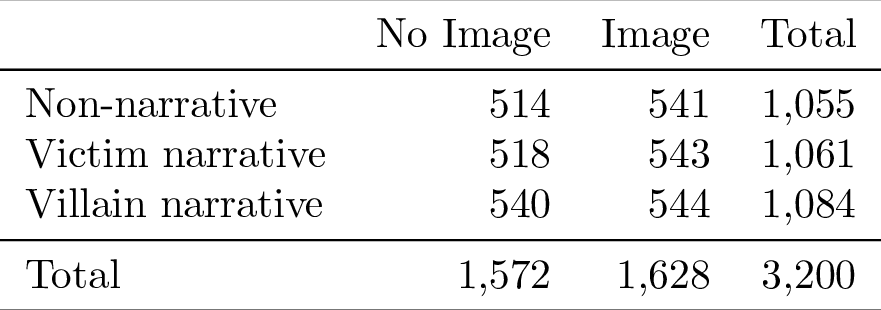

Supplement: S1 Table — (TIF) [file pone.0244440.s011.tif]

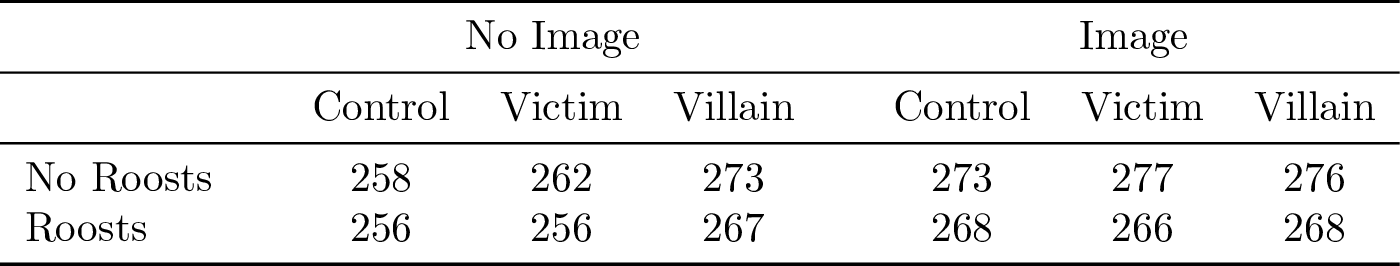

Supplement: S2 Table — (TIF) [file pone.0244440.s012.tif]

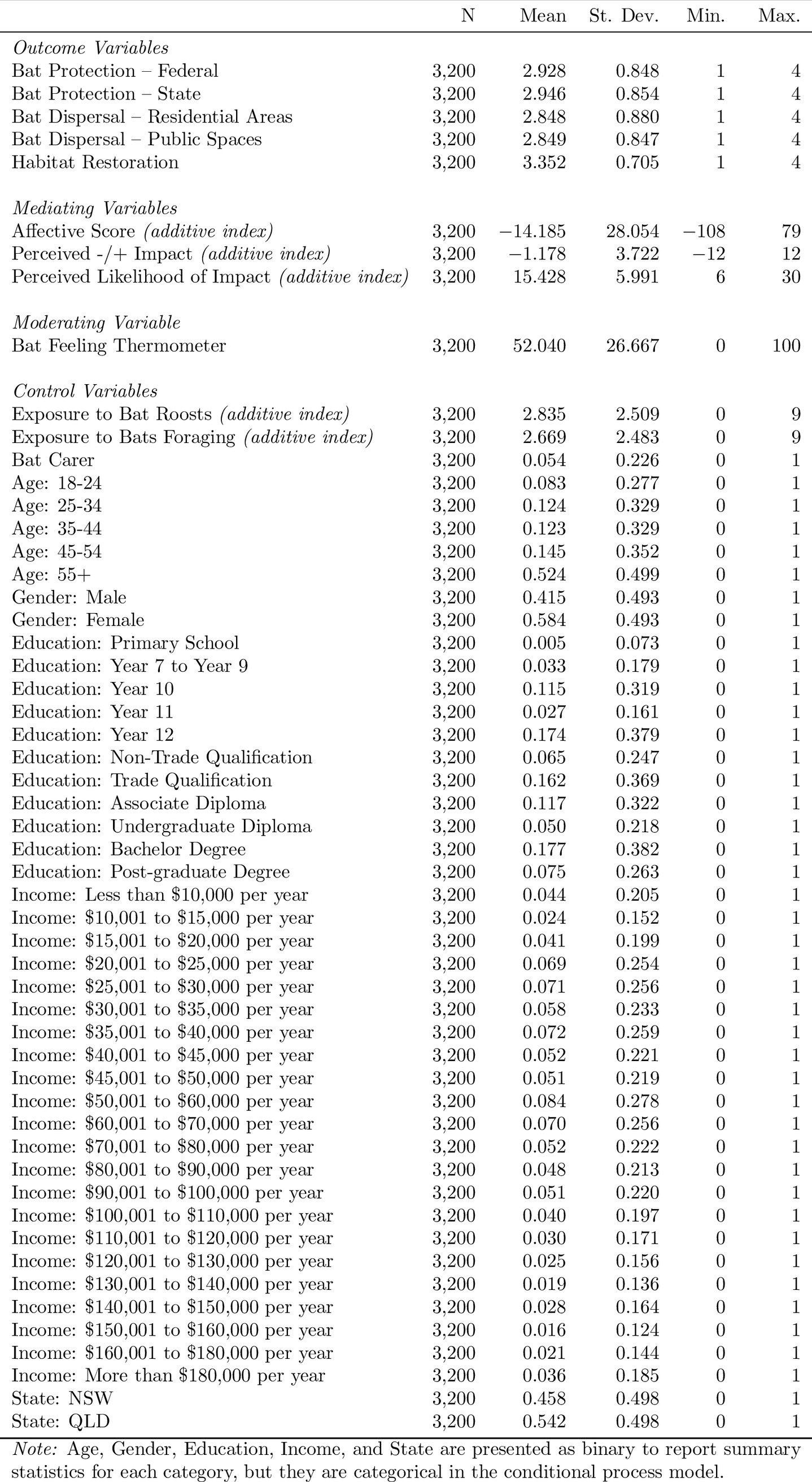

Supplement: S3 Table — (TIF) [file pone.0244440.s013.tif]

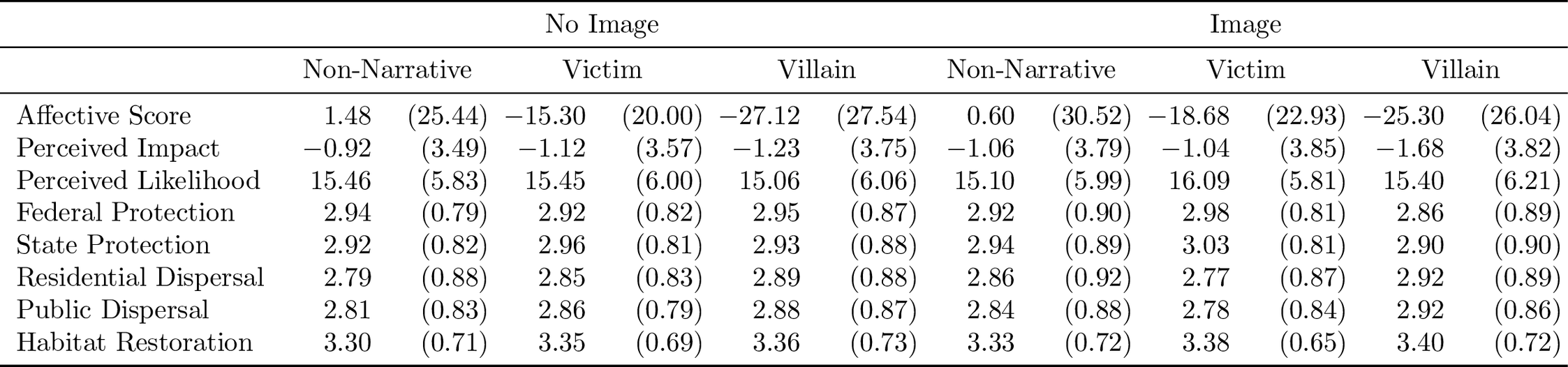

Supplement: S4 Table — (TIF) [file pone.0244440.s014.tif]

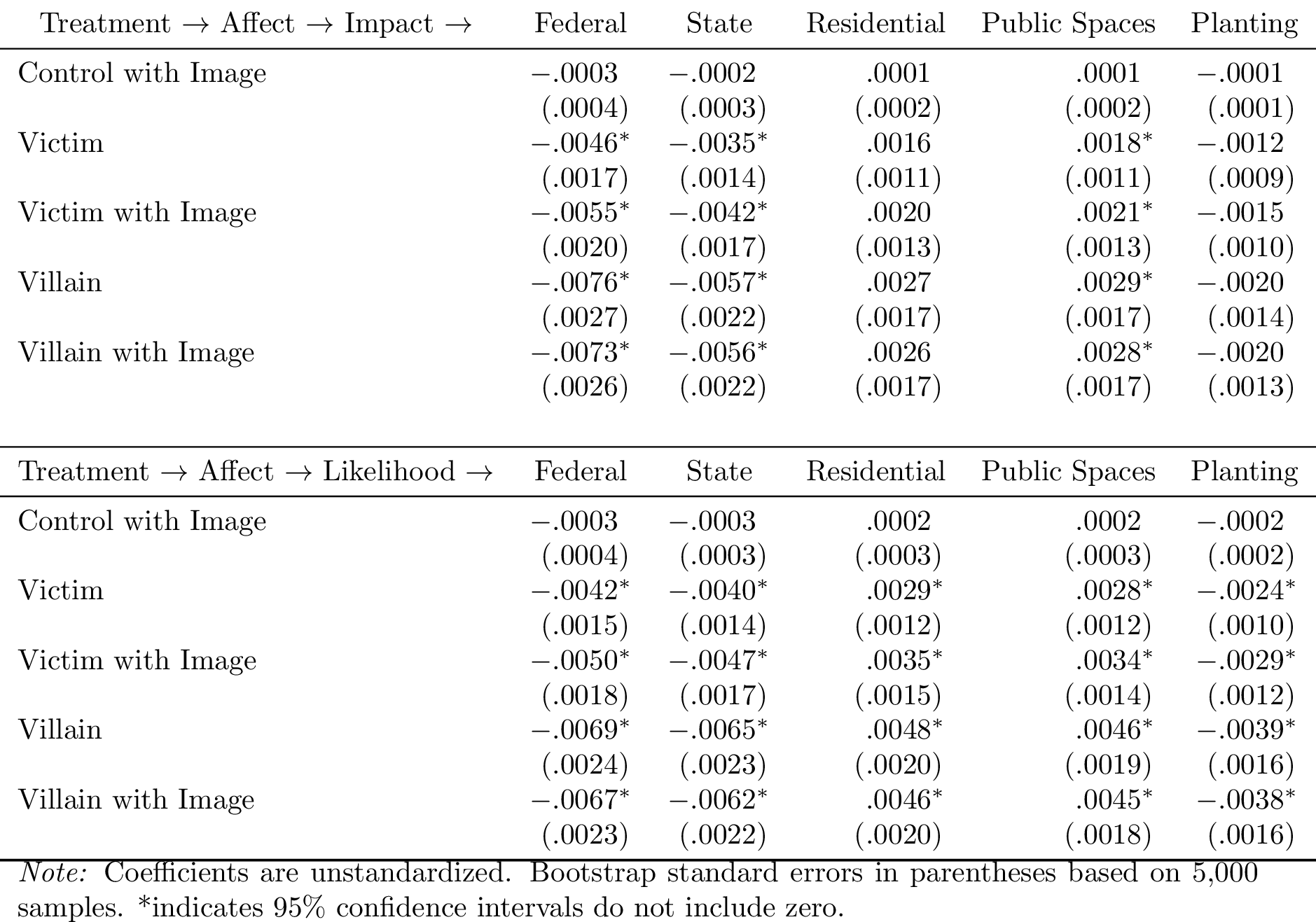

Supplement: S5 Table — (TIF) [file pone.0244440.s015.tif]

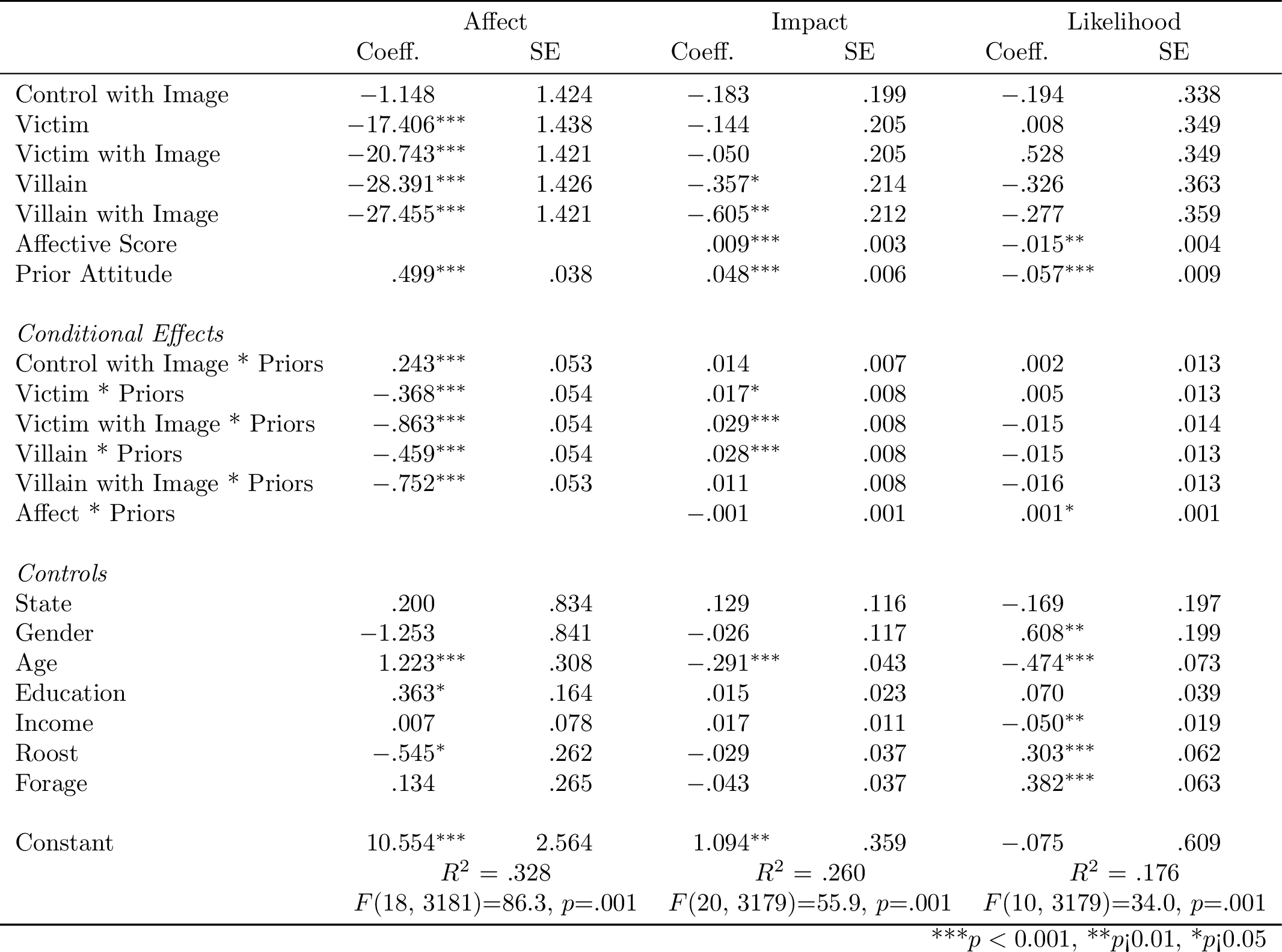

Supplement: S6 Table — (TIF) [file pone.0244440.s016.tif]

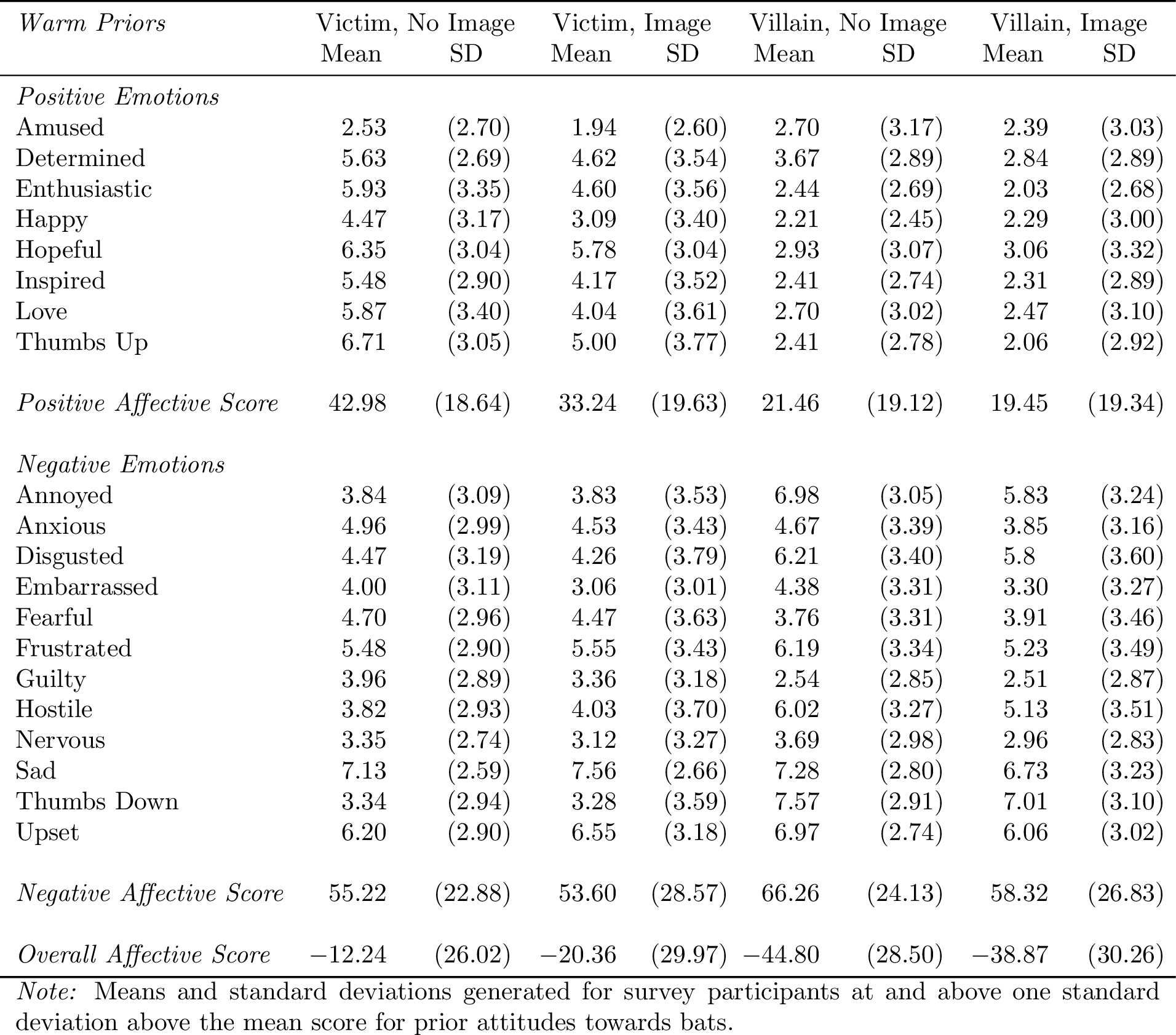

Supplement: S7 Table — (TIF) [file pone.0244440.s017.tif]

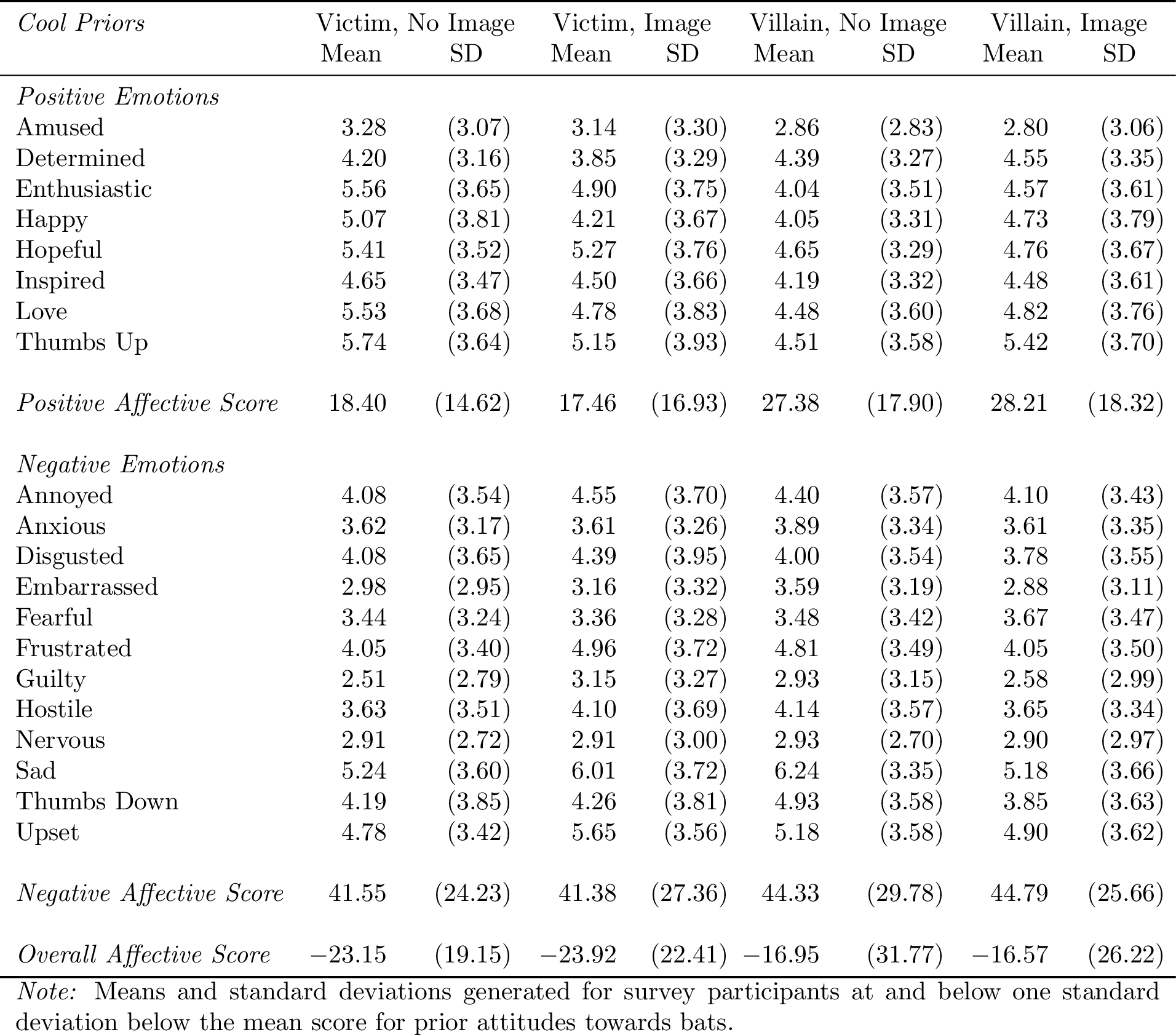

Supplement: S8 Table — (TIF) [file pone.0244440.s018.tif]

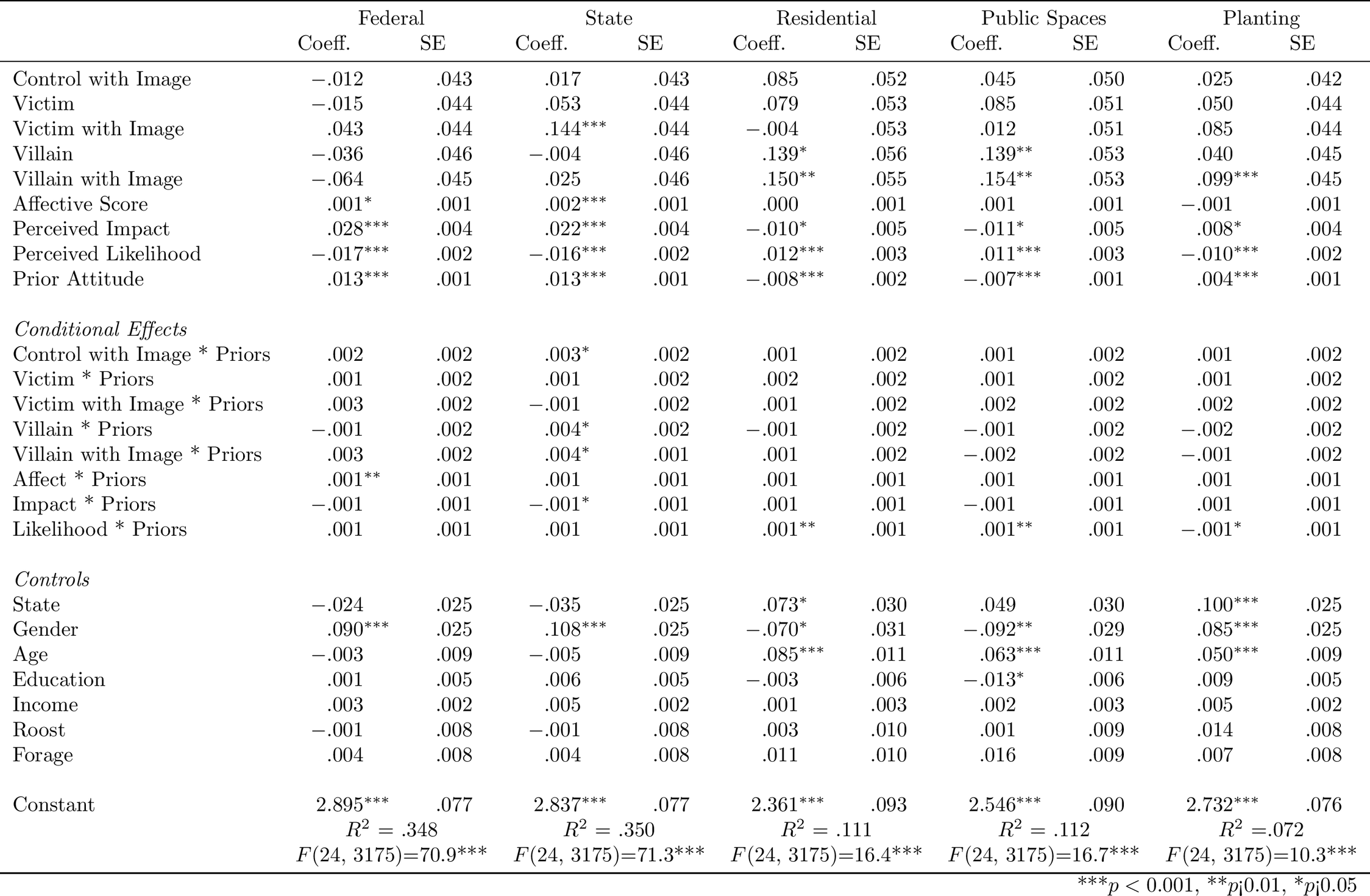

Supplement: S9 Table — (TIF) [file pone.0244440.s019.tif]
