# Supplementary material for: Communicating risk in human-wildlife interactions: How stories and images move minds
Source: PLoS One. 2020 Dec 28;15(12):e0244440. doi: 10.1371/journal.pone.0244440 (PMC7769453; doi:10.1371/journal.pone.0244440)
Supplement: Attachment — Submitted filename: Response Memo.pdf [file pone.0244440.s020.pdf]

To: Editor and Referees, *PLOS ONE*

From: Sara K. Guenther and Elizabeth A. Shanahan

Subject: Revise and Resubmit, *Communicating risk in human-wildlife interactions: how stories and images move minds*

Date: December 4, 2020

This memo documents the revisions made to the manuscript in response to feedback from the Editor and Referees. The changes motivated by the Editor's and Referees' comments have no doubt made the paper stronger. Thank you for your time and consideration.

Below, we respond to each point of feedback individually. We note the reviewer suggestions as bullet points and detail our revisions in response. Where appropriate, we include line number references corresponding to the revised manuscript (without track changes).

Editor

- "...I am receptive to the point that made here (i.e. that narratives should be used for conservation communication), yet my concern is that it cuts both ways. If we start advocating for "narrative" messages in favor of bats, the scientific community (and to a larger extent the conservation community) will not be legitimate anymore in arguing against the same methods being used by adversaries. Currently, we are still in a position in which we can oppose the latter by stating that what we offer to the public is evidence-based knowledge, devoid of political aims, subjective feelings or "narratives". As somebody who has communicated extensively in the media about bat-farmer conflicts (esp. regarding Mauritius), I reckon that this stance is precious to convince the public. Deviating from this line and starting to promote and spread narratives might open a Pandora box. This really needs to be somehow acknowledged in your manuscript I think. I will not request for a solution to this complex problem be detailed (this could be the object of another self-standing paper), but I feel that a bit of discussion is needed to inform the readership about the risks. Furthermore, I reckon that you have been very careful in your wording and recommendations. But as your message will be (mis)interpreted, I think that you might want to prevent your recommendation to be seen as a call for the use of propaganda-like communication in conservation."

Thank you for this important and thought-provoking comment, we are certainly sympathetic to the concern raised here. From our reading, the central concern is that scientists would lose credibility with the public by using narratives that favor bats to communicate scientific information.

We'd ask you to consider three relevant points. First, our narrative treatment conditions were designed based on the type of communication we observe presently and

persistently about flying foxes on social media among the general public (e.g., citizens, advocates, politicians, celebrities) – reflecting both positive and negative perceptions and portrayals. The primary aim of our paper is to understand the effect of the proliferation of this type of messaging – which is already circulated – within and between social networks. Our experimental design incorporated treatments to mimic, to the best of our ability, the social media environment and test the downstream effects of this type of communication on affective response, risk perception, and public opinion. While our work does have implications and could offer guidance for communicators in the sciences and across stakeholder groups, first and foremost we are interested in measuring and reporting the effects of narrative communication similar to what we already observe. Germane to this point is that the messenger (the Facebook account posting the message) does not vary across conditions and is in fact fictitious/anonymous/censored.

A future experiment might explore the very question posed in the Editor's comment, also raised in Dahlstrom 2014 [1]. Does narrative communication affect scientific credibility and/or trust in scientists who weigh in on human-wildlife conflict? The design might feature the same scientist communicating in non-narrative form to a control group and in a narrative form to a treatment group, and participants might be polled afterwards on the credibility of the messenger.

Second, in terms of applicability of the findings in this paper to future communication efforts, scientists are not the only potential messengers; in fact, we had government officials and advocacy groups in mind when considering the implications of our findings. These actors may not be held to the same standard of objectivity as scientists in the public eye.

Third, though the narrative messages portrayed in our treatment conditions were based on our observations of actual discourse about bats on social media, we are also interested in contributing to a larger body of work on *effective* risk communication. This paper suggests that risk (to bats or to humans) communicated in narrative form is compelling, and that images accompanying narrative risk communication are received differently based on a person's prior attitudes, which is a good first step. Another paper might test whether communicating risk in narrative form increases precision in the perception of *actual* risk, assuming an ex ante discrepancy between public's perceived risk (e.g., contracting *Lyssavirus* from bats) and actual risk.

We do appreciate the Editor's concern, and wish to address it in the manuscript directly. Therefore, we added the following text to the Discussion:

> While our work may provide insight for communicators in the sciences and across stakeholder groups, we urge careful consideration of the ethical questions raised in the practice of narrative risk communication, including a thorough examination of the goals (e.g., persuasion, comprehension) and attention to the level of accuracy maintained [(Dahlstrom 2014)]. Science communicators may want to pay special attention to issues of trust and credibility, and future work ought to evaluate the impacts of narrative risk communication on messenger credibility. Reasonable hypotheses offer competing predictions: narrative communication may increase trust in communicators through perceived authenticity and accessibility, or decrease trust from perceived intention to manipulate [(Dahlstrom 2014, Dahlstrom and Rosenthal 2018)]. [Lines 463-472]

- "The case that you studied here (FFs in Australia) is very interesting and valuable. You might want to show that it is illustrative of a global issue, as flying foxes are persecuted and threatened all over their range. See https://science.sciencemag.org/content/355/6332/1368 and references therein."

We have added a section, "Case description," which more clearly explains why we have chosen to examine the case of flying foxes in Australia; to address the Editor's comment specifically, we have included the following text and references in this section: "Similar strained relations between humans and flying foxes have been noted all over their range of distribution, contributing to individual and collective management decisions that threaten the longevity of these species (e.g., mass culling, destruction of roosts) [(Vincenot 2017, Musila 2018)]" [Lines 140-143].

- "Similarly, there were few studies on perception of flying foxes, which adds to the relevance and importance of your work. You may want to stress this by citing the few existing works (and perhaps discuss some of their results compared to what you observe here). See for instance: https://link.springer.com/article/10.1007%2Fs10745-017-9905-6;https://www.sciencedirect.com/science/article/pii/S2351989415000190"

We have added a paragraph summarizing previous studies on the perception of flying foxes [Lines 147-159], and have added the references recommended by the Editor. In particular, we show that existing work documents both negative and positive perceptions of bats. Though negative perceptions are more thoroughly documented, studies do show a rising trend in positive perceptions correlated with higher education. The variation in attitudes towards flying foxes supports our selection of the case since one of our aims is to understand how prior attitudes condition effects of narrative risk messaging. Furthermore, we have identified a few prior studies that document social media as a forum for sharing and disseminating negative and positive perceptions publicly, an observation that motivated the design of our experimental treatment, and we have added those references as well (Fagan *et.al.* 2018, López-Baucells *et.al.* 2018). Most importantly, a survey of prior studies reveals that the mechanisms mediating the effect of messaging and imagery on support for conservation efforts have not been evaluated prior to our study.

- "Minor, but 'mega bat' should be spelled 'megabat'. (Please note that, although still used in common language, it is actually considered now better replaced by "Old World fruit bat'", or more strictly, Yinpterochiroptera/Pteropodid, in scientific publications.)"

We have clarified our reference to flying foxes by removing the term "megabat" in favor of the following: "Flying foxes, also known as Old World fruit bats (genus *Pteropus*), serve as critical pollinators for Australia's forests" [Lines 130-31].

R1

- "Intro – previous attitudes and mechanism of change – please explicitly speak about congruent or incongruent change"

To expound on how scholars have previously conceptualized prior attitudes as mechanisms of change, we added the language suggested by the reviewer, with a corresponding citation, to our review of current literature on prior attitudes and mechanisms of change. See Lines 69-72.

- "Final para [of Intro] – it is not clear why bats were chosen, please write short para about public perception of bats and finish the para with argument(s) that bats are perfect examples of human-wildlife conflict."

As mentioned above in response to the Editor's comments, we have added a paragraph about public perception of bats [Lines 147-159]. Please also see our response to the Editor above.

- "[Line 104] — see e.g., Musila, S., Prokop, P., & Gichuki, N. (2018). Knowledge and perceptions of, and attitudes to, bats by people living around Arabuko-Sokoke Forest, Malindi-Kenya. Anthrozoös, 31(2), 247-262."

Thank you for drawing our attention to this study; we have added this reference to the text [Lines 7, 143, 149].

- "[Line 267] — negative perception of animals = low conservation support, see Gunnthorsdottir, A., 2001. Physical attractiveness of an animal species as a decision factor for its preservation. Anthrozoös 14, 204–215; Prokop, P., & Fančovičová, J. (2017). Animals in dangerous postures enhance learning, but decrease willingness to protect animals. Eurasia Journal of Mathematics, Science and Technology Education, 13(9), 6069–6077.

We have added both recommended references to the text (Gunnthodsdottir: Lines 56, 60; Prokop: Line 60). We also added another Prokop reference in Line 148 [2].

- "A figure with visual presentation of villain and victim treatment is required."

We updated Figure 2 to include the non-narrative and the villain narrative conditions alongside the victim narrative. We also consolidated the supplementary figures depicting narrative messages without images into one figure.

R2

- "I think you could draw clearer theoretical implications by exploring (in the literature review and discussion) the theoretical mechanisms of the effects, but I do not see that as essential. "

We have added the following text in the literature review to clarify our theoretical implications: "In this study, we develop an inclusive, social science model by incorporating three theoretically-based concepts in the realm of risk communication: risk perception, affect heuristic, narratives, and prior beliefs" [Lines 15-17].

- "Perhaps there's another way to phrase "human-flying fox conflict," which may easily refer to a conflict about foxes that fly humans...You might also highlight in the abstract that flying foxes are a type of bat. Of course they are, but some readers might not know that."

Thank you for bringing this to our attention. We have removed the phrase "human-flying fox conflict" from the Abstract and use the following wording instead: "Using a survey experiment fielded in the midst of contentious public debate over flying fox management in urban and suburban areas of Australia..." In the remaining part of the Abstract, we are consistent in the use of the terms "bat" or "bats" when referring to flying foxes to avoid confusion. In the main text, we refer to "human-bat conflict" (as opposed to "human-flying fox conflict") once, in Line 372.

- "You have a parenthetical aside, 'severity/cost * likelihood,' which creates ambiguity over the use of the forward slash."

We have revised the phrasing to: "severity of impact x likelihood of impact" [Line 21].

- "You cited Green and Brock to raise the concept of transportation. I wish to call your attention to Dahlstrom (2014; https://doi.org/10.1073/pnas.1320645111), who did a nice job discussing the influence of narratives in the contexts of science and environmental communication. I am not sure your citations 25 through 28 (narratives in risk contexts) included comparisons of narrative and non-narrative texts. If they do not, you might cite another one of Dahlstrom's works, which made that comparison in the context of climate change denial (see Dahlstrom and Rosenthal, 2018; https://doi.org/10.1177/1075547018766556)."

Our references (formerly 25-28, now 32-33 and 35-36) are studies that compare narrative and non-narrative communications. However, we find Dahlstrom's 2014 overview to be an excellent addition and have added it to our literature review, as well as to the Discussion [Lines 52, 385]. We have also added the Dahlstrom and Rosenthal 2018 reference (citation 34 in the manuscript) in Line 54.

- "There has also been recent work in the environmental communication arena looking at the linkages between positive and negative affect and benefit and risk perception (e.g., Kahlor et al., 2019; https://doi.org/10.1080/17524032.2019.1699136). That work seems germane to the point you make after mentioning transportation. "

We have added the Kahlor reference with the following text:

> Additionally, the import of affect in communication rests on the concept of transporting the audience through an affective experience [(Green and Brock 2000)]. **Indeed, modeling both positive and negative affect, as well as perceived benefits and risks, has deepened our understanding of information-seeking behavior in other areas of environmental communication [(Kahlor *et.al.* 2020)]**. Therefore, to advance a more complete representation of the HWCC system, the range of positive to negative affect needs to be recognized... [Lines 37-42]

Though we added the Kahlor reference to the text, we do note that the Kahlor study models perception of benefits and risks as occurring before affective response (e.g., worry, hope). This deviates from our model of affective response before cognition, which is supported by the work of Brock and Green, Green and Brock, and Kahneman, all of whom posit that affective responses occur before cognitions.

- "You should clarify early on that you are portraying bats, not humans, as villains. This was unclear until the research design. You also need to clarify the source of positive and negative impacts. When I initially read your series of predictions, I inferred a counter-intuitive mediation model in which the portrayal of victims leads to positive emotion, which leads to positive perceived impacts (on the victims). I came to understand you meant the impact of the victims on humans, but only after reviewing S1."

While we detail the character assignment in the Research Design section where we describe each treatment condition, following the Reviewer's suggestion we add a clarification of character assignment in villain/victim narrative earlier in the text, in our series of predictions at the end of the Introduction and before Case Description:

> [W]e anticipate that narrative casting wildlife as villains results in higher negative affective response (e.g., frustrated, upset, disgusted), while narrative casting wildlife as victims results in higher positive affective response (e.g., hopeful, inspired, determined). [Lines 110-113]

We also made adjustments to the text to clarify the source (wildlife) and object (humans) of positive and negative impacts:

> [W]e expect a more negative affective response to engender more negative perceived impacts of wildlife on humans, while a more positive affect will engender more positive perceived impacts of wildlife on humans. We predict that the intensity of affective response, as opposed to whether affect is positive or negative, will determine the perceived likelihood of impacts on humans. Finally, we expect negative and likely perceived impacts on humans to predict more support for wildlife relocation, and positive and likely perceived impacts to predict more support for wildlife protection. [Lines 115-122]

- "Please clarify how prior attitudes will condition the effects of narratives and images. You clearly indicated a positive moderation effect of images on the effect of narratives. What kind of effect do you anticipate for attitudes and how does that reflect extant scientific knowledge?"

Thank you for this comment. Adding the paragraph summarizing prior literature on public perceptions towards flying foxes [Lines 147-159] has provided useful context that clarifies our expectations with respect to the role of prior attitudes in conditioning causal pathways from narrative to policy support. We have also followed your suggestion elsewhere vis a vis organization of our expectations in accordance with the conceptual stages of the model. As such, we have also clarified our expectation regarding moderating effects here:

> As for moderating effects, we expect prior attitudes towards wildlife to condition the strength of these relationships such that warm prior attitudes correspond with more favorable responses and perceptions towards wildlife, while cool prior attitudes correspond with less favorable responses and perceptions towards wildlife. [Lines 122-126]

- "In my summary comment, I stated that the theoretical contributions are lacking. I wish to give you a brief example. On page 4, you write, 'Understanding the principal mechanisms that exacerbate human-wildlife conflict is crucial to the development of successful policies...' Then you identify the 'Narrative Policy Framework' as a theoretical anchor. However, you do not explain any of the mechanisms of that framework. In fact, that is the only instance of 'Narrative Policy Framework' in your manuscript (excluding the references)."

Thank you, we have added the following text:

> First, we use the theoretical anchor of the Narrative Policy Framework (NPF) [(Shanahan *et.al.* 2018)] to inform the structure of the narrative risk message treatments. **The NPF posits that narratives are measurable across policy domains, because narratives themselves have a reliable structure that includes elements such as characters, setting, and plot [(Shanahan *et.al.* 2013)]**. As such, we can isolate narrative mechanisms (i.e., humans or wildlife portrayed as villain or victim) to more precisely and reliably test their effects. [Lines 80-91]

- "The two paragraphs about flying foxes seem to be about the context of your study, which is not clearly about the research design. Perhaps you could put them in a dedicated 'context' section. I think that would go well at the end of the literature review."

We have added the subheading, "Case description," and separated it from the "Research Design" section.

- "Based on your Figure 1 note, I presume all direct and indirect paths were moderated by W. You should add 'W' in parentheses as you do with $M1$, $M2$, $M3$ and Yj, and locate it immediately after 'prior attitude towards bats.' I think that will help the reader understand your implementation of PROCESS."

We have revised the Figure 1 caption based on this suggestion.

- "Your first statement implies overall support for your predictions about narratives and images. You had predicted 'the use of an image with narratives will intensify affective response.' Table S4 does not appear to show that. It appears after adding the image, the victim condition resulted in a more negative affective score and the villain condition resulted in a more positive affective score. I suspect those differences are significant, which would run counter to your prediction, no? I would like to see more discussion of the treatment effects on affective score, which would help make sense of the subsequent effects. I understand the treatment effects are conditioned on priors, but your prediction in the literature review first indicates the unconditioned treatment effects before regarding the conditioning by priors."

We agree with the reviewer that this finding deserves a clearer explanation with respect to our expectations. We add the following text to our discussion of the results:

We expected the addition of an image alongside narratives to intensify affective response, but found that this was only true some of the time and not always in the direction we anticipated. An image of flying foxes presented alongside a victim narrative intensified a negative affective response; but an image presented alongside a villain narrative dampened, or reduced, the negative affective response. This pattern is reflected in a comparison of the means across conditions (S4 Table) and in the comparative magnitudes of the indirect effects (S5 Table). [Lines 227-234]

- "Your analysis involves, essentially, a multiple-moderated mediation of a 2 x 3 factorial treatment effect. This is a complex analysis, not from a technical standpoint, but from how best to present it to readers. I think you have done generally a good job focusing the analysis on a few key findings. However, I wonder if there is a more structured organizational approach. For example, when you state (at the end of the literature review) the findings you expect to see, you might structure it in the three-stage format of your results."

We have added text and restructured the statement of our expectations to better match the structure of the conceptual model and Results section. See Lines 99-126.

- "Several of the observed effects are very small ($R^2 < .01$). I hope you can add a note in the discussion about the practical significance of those findings."

The reviewer is likely referring to the reported difference in R^2 between an unconditional model and conditional model, our evaluation of the moderating effects of prior attitudes [3]. The change in R^2 in the first stage, between treatment and affect, is 0.14 [Line 255]. Another way to interpret this is: 14% of the variance in affective response is attributable to the moderating effect of prior attitudes towards bats. Or, the inclusion of an interaction between treatment condition (X) and prior attitudes (W) in the model leads to a 14% increase in R^2.

The change in R^2, or the moderating effect, is largest in the first stage of the model, which suggests that prior attitudes may be more important in conditioning affective response to risk communication and less important further down the path to policy support. Though we do find statistical significance for the moderating effect of prior attitudes in subsequent stages in the path, the reviewer is correct that these effect sizes are considerably smaller. For example, the moderating effect between affect and perceived likelihood amounts to a 0.1% increase in R^2, which is statistically significant, but substantively quite small compared to 14%. Additionally, we found a statistically significant 0.1% increase in R^2 between perceived impacts of bats and support for bat protection, a 0.2% increase in R^2 between perceived impacts of bats and support for bat dispersal, and a 0.2% increase in R^2 between perceived likelihood of impacts and support for habitat restoration. In comparison, the R^2s we report in Tables S6 and S9 range from 0.35 (35%) to 0.07 or (7%), with an average R^2 of 0.22.

We address the reviewer's comment in the manuscript text as well, by adding the following text:

> It is worth noting that survey participants' prior attitudes towards bats had the largest effect in the first stage, affective response to treatment conditions, compared to subsequent stages on the path of mediation. We found

the moderating effect of prior attitudes towards bats to be comparatively smaller in predicting variation in risk perception and support for policies. This suggests that prior attitudes influence initial receptivity and reactivity to certain types of messaging more than influencing the way affective response translates to perceived risk, and the way perceived risk translates to support for policies. [Lines 441-448]

- "You invoke the concept of transportation in explaining the effect of narrative. However, I am not convinced transportation was the mechanism. Why not something simpler like involvement? Do you believe the brief narratives contained enough story to create a transporting effect? If so, why? And then what does the addition of images mean for your theoretical account? Green describes transportation as an 'integrative melding of attention, imagery, and feelings.' What happens when the narrative provides the images for the reader?"

The way we set up the model, we expected the effect of narratives on policy support to be mediated by affect. Our results largely support this expectation. We allow prior attitudes to condition the effects in the model, and also control for exposure (proximity to roost and forage) and geographic location/institutions (state). (More detailed description of covariates is available in the "Supporting information" section.) Therefore, we believe we adequately account for several dimensions of involvement in our modeling, and are convinced of our use of affective response as a proxy for transportation. Additionally, the reviewer wonders whether the length of the treatments would be enough to create transportation effects. This is an excellent question, and we maintain that transportation can occur with the briefest of narratives and with images (e.g., memes or sentences out of religious texts or inspiring message calendars). Theoretically, images in conjunction with narrative text would heighten emotion, add to the transportation effect, and amplify the persuasiveness of narrative messages. We approached images as another dimension of transportation that would add cumulatively to the effect of narratives; our findings, however, suggest that this heightening effect of images is conditioned on prior attitudes toward the subject. (Here we refer back to our response to the comment above about clarifying our results vis a vis our expectations about the addition of images.)

References

[1] Dahlstrom MF. Using narratives and storytelling to communicate science with nonexpert audiences. Proceedings of the National Academy of Sciences. 2014;111(Supplement 4):13614–13620.

[2] Prokop P, Fančovičová J, Kubiatko M. Vampires are still alive: Slovakian students' attitudes toward bats. Anthrozoös. 2009;22(1):19–30.

[3] Hayes AF. Introduction to mediation, moderation, and conditional process analysis: A regression-based approach. Guilford publications; 2017.
